# Decoding Spatial Memory Retrieval in Cubical Space Using fMRI Signals

**DOI:** 10.3389/fncir.2021.624352

**Published:** 2021-05-18

**Authors:** Jiahe Guo, Kai Zhang, Jianyu Zhang, Rui Zhao, Yibo Liang, Yu Lin, Shengping Yu, Wen Qin, Xuejun Yang

**Affiliations:** ^1^Department of Neurosurgery, Tianjin Medical University General Hospital, Tianjin, China; ^2^Department of Surgery, First Teaching Hospital of Tianjin University of Traditional Chinese Medicine, Tianjin, China; ^3^Graduate School, Kunming Medical University, Kunming, China; ^4^Department of Orthopaedic Surgery, Tianjin Medical University General Hospital, Tianjin, China; ^5^Department of Otorhinolaryngology Head and Neck Surgery, Tianjin First Central Hospital, Tianjin, China; ^6^School of Medical Imaging and Tianjin Key Laboratory of Functional Imaging, Tianjin Medical University, Tianjin, China; ^7^Department of Neurosurgery, Tsinghua University Beijing Tsinghua Changgeng Hospital, Beijing, China

**Keywords:** spatial memory retrieval, cubical space, GLM, MVPA, occipitoparietal cortex, right middle temporal gyrus, task-based fMRI

## Abstract

The way spatial memory retrieval is represented in the brain remains unclear to date. Previous studies have displayed a hippocampus-centered navigation network using functional magnetic resonance imaging (fMRI) analysis. There have been some studies on the representation of navigation behavior by signal distribution patterns, but only in the hippocampus and adjacent structures. In this study, we aimed to determine (1) the brain regions that represent information in both intensity and distribution patterns during spatial memory retrieval and (2) whether the patterns of neural responses represent spatial memory retrieval behavior performance. Both univariate analysis [general linear model (GLM)] and multivariate pattern analysis (MVPA) were employed to reveal the spatial distributions of brain responses elicited by spatial memory retrieval. Correlation analyses were performed to detect the correspondences between brain responses and behavior performance. We found that spatial memory retrieval occurred in widespread brain regions, including the bilateral hippocampi, bilateral superior frontal gyrus, bilateral superior parietal lobules, bilateral occipital lobes, and cerebellum. The amplitude of activation in the left hippocampus showed a significant negative correlation (*r* = −0.46, *p* = 0.039) with the number of task completions. Additionally, within-subject classification accuracies based on the blood oxygenation level-dependent (BOLD) signal patterns of the right middle temporal gyrus (rMTG) rostral areas in the Brainnetome Atlas showed a significant positive correlation (*r* = 0.78, *p* < 0.0001) with retrieval accuracy. In summary, our findings have implications for understanding the separation between navigational and non-navigational states and emphasizing the utility of MVPA in the whole brain.

## Introduction

Spatial memory is the storage of information about orientation and location. The way spatial memory is represented in the brain has been a motivating question for decades (Eichenbaum et al., 1999; Buzsaki and Llinas, 2017; Bellmund et al., 2018). Tolman first conducted a water maze experiment in mice to probe this exciting issue and concluded that spatial memory was represented in the central nervous system rather than being a simple stimulus response (Tolman, 1948). This finding was further demonstrated by O’Keefe and colleagues’ discovery of place cells in the rodent hippocampus, which allowed neural instantiation of spatial location in the central nervous system (O’Keefe and Dostrovsky, 1971; O’Keefe and Nadel, 1978). Although correlations between small numbers of neurons and spatial location have been delineated in animals, they have not yet been mapped in the complex human nervous system. However, depicting the nervous activity of spatial memory on a macroscopic scale is essential (Hassabis et al., 2009).

Forty years later, a self-paced Roland’s hometown walking task was initially designed and applied to evaluate mental activity changes involving long-term spatial memory retrieval using positron emission tomography–computed tomography (PET–CT) (Roland et al., 1987). Since then, Roland’s hometown walking task has frequently been used in neuroimaging studies to assess patients’ spatial memory function (e.g., anterior temporal lobe resection, temporal lesions) (Avila et al., 2004, 2006; Janszky et al., 2005; Bonelli et al., 2010; Strandberg et al., 2017). Despite congruent findings, such a free-behaving experiment remained a niche due to uncontrollability and unnecessary complexity, resulting in essentially uninterpretable data (Maguire, 2012). Hence, the correspondence between the behavior performance of memory ability and neural responses is a challenging area to investigate (Banta Lavenex et al., 2015; Fernandez-Baizan et al., 2019a, b). To overcome the limitations mentioned above, investigators have proposed numerous paradigms to quantify spatial memory retrieval process by two-dimensional space, such as recognition visual field location (Jeye et al., 2018; Fritch et al., 2020) or scenario pictures (Lacy et al., 2011), and three-dimensional space, such as a virtual round arena (Nilsson et al., 2013), spatial route learning (Rekkas et al., 2005), the virtual Morris water maze (Antonova et al., 2011; Reynolds et al., 2019), and perspective changing in space (Lambrey et al., 2012). Notably, these previous works frequently used univariate statistical models, focusing on the hippocampus and entorhinal cortex (EC) functions in spatial memory and navigation processes. Neuroimaging and neuropsychological studies have indicated that the hippocampus and EC are not the only regions that mediate spatial memories. The pre-existing spatial cognitive map in some patients remains intact after medial temporal lobe (MTL) impairment (Teng and Squire, 1999), consistent with our clinical experience in patients who underwent MTL resection. In addition, the univariate method may have overlooked information beyond the amplitude of the brain signals (e.g., spatial patterns of brain activation). Spatial signal distribution deserves attention due to the complexity of the cognitive spatial map in the brain. The bridge between the MTL and other cortices associated with spatial memory has not yet been fully built (Maviel et al., 2004; Vann and Albasser, 2011; Moscovitch et al., 2016; Sekeres et al., 2018).

To analyze the distribution pattern of brain signals detected by functional magnetic resonance imaging (fMRI) or electroencephalogram (EEG), multivariate pattern analysis (MVPA) has received increasing attention in brain imaging data analysis (Hassabis et al., 2009; Chadwick et al., 2010, 2012; Bonnici et al., 2012; Nielson et al., 2015; Deuker et al., 2016). MVPA can detect effective information in high-dimensional data to a greater extent, discriminating between experimental conditions and searching for slight individual differences to prevent signal-loss issues (Pereira et al., 2009). Cognitive maps formed in the MTL have been identified in a few studies using MVPA based on fMRI signal patterns. Dissociated patterns between responses in the hippocampus and parahippocampal gyrus were observed in Hassabis and colleagues’ research with two virtual squared rooms (Hassabis et al., 2009). Subsequent work with a large-scale quasi-real environment confirmed that spatial and temporal events could reflect the similarity of neural patterns in the hippocampus from different subjects (Deuker et al., 2016). Another study showed various activation patterns in the anterior hippocampus during retrieval of one’s life, representing distinct experience integration in reality (Nielson et al., 2015). Undoubtedly, neural activation within the MTL can provide a scaffold for episodic memory and navigation, whether in a real or a virtual world. Nevertheless, few studies have used MVPA to study spatial memory representations within the navigation network or brain level.

In the present study, a well-designed three-dimensional spatial memory retrieval task with an egocentric view (Hassabis et al., 2009) was used, and blood oxygenation level-dependent (BOLD) signals were collected. Both univariate and multivariate methods were conducted to identify the brain regions encoding the spatial memory retrieval process, and correlation analyses were performed to reveal the correspondence between neural activities and the behavior performance of memory retrieval. We aimed to determine (1) the brain regions representing the information in both intensity and distribution patterns during spatial memory retrieval and (2) whether the patterns of neural responses reflect a person’s spatial memory ability.

## Materials and Methods

### Subjects

Twenty young healthy volunteers (men, ranging from 20 to 30 years, 26.3 ± 3.6 years) with no history of psychiatric or neurological illness were recruited. All participants were right-handed, as ascertained by the Edinburgh Handedness Inventory. The study was approved by the ethics committee of Tianjin Medical University General Hospital. All the participants provided written informed consent before the study.

### MRI Data Acquisition

All MRI data were acquired using a 3.0 T magnetic resonance scanner (Siemens Prisma) with a 64-channel phased-array head coil. Functional data were acquired using a simultaneous multislice, gradient echo, echo-planar imaging sequence (EPI) with the following parameters: echo time (TE) = 30 ms, repetition time (TR) = 750 ms, field of view (FOV) = 222 × 222 mm, matrix = 74 × 74, in-plane resolution = 3 × 3 mm, flip angle (FA) = 54°, slice thickness = 3 mm, no gap between slices, number of slices = 48, and slice orientation = transverse.

### Experimental Design

**FIGURE 1:**
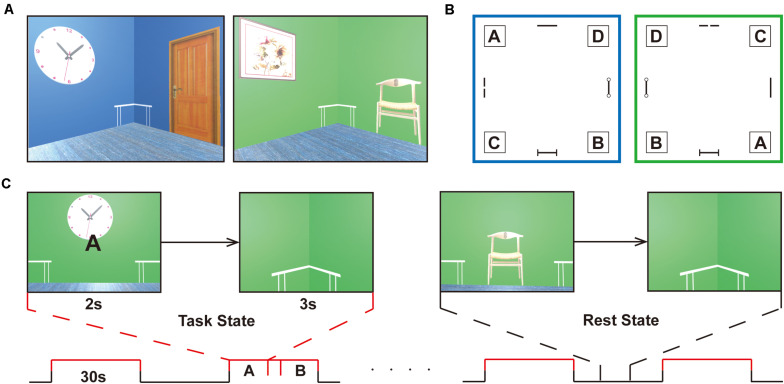
Experimental design. **(A)** Navigation task is completed in two virtual reality rooms of different colors (i.e., green or blue) with four decorations on the wall (i.e., clock, panting, door, chair). **(B)** Schematic of the room layouts with the four target corners, labeled A, B, C, and D. A birch-colored table is placed in each corner of the room and is immaterial as a cue during navigation. The target positions are set under the tables, corresponding to the letters, A, B, C, and D. The two rooms are designed to increase the difficulty of the task. **(C)** The task proceeds in a block-design manner with a 30 s duration of each block. The task stage alternates with the resting stage in the course of 8 min. In the retrieval stage (task state), participants enter the room and face the wall, where a random letter for the navigation target is displayed for 2 s. When they arrive at the targets, the viewpoint transitions downward so that the identical floor texture occupied the entire field of view. Participants will be given a feedback (a check mark or a cross) after navigation in the learning and testing phase, but not in the scanning stage. After a 3 s countdown, a new letter was given that was to be followed. In the non-retrieval stage (rest state), no letter prompts and no movements were made, and random perspectives were displayed. The subjects were requested to press buttons at the same frequency as the task and watch the screen to ensure visual matching. The entire task is only used to distinguish between navigational (task) and non-navigational (rest) states.

A validated three-dimensional spatial memory task was applied to evaluate the memory retrieval of spatial locations (Hassabis et al., 2009). All experimental tasks were implemented with the Unity game engine^1^. An MRI-compatible response collection system with four buttons was available for fMRI experiments, which allowed participants to freely move forward or backward and turn right or left through the environment. During the navigation task, subjects were required to follow a given letter to a specific location in two virtual reality rooms. Each room was 8 × 8 m in square and 3 m in height and consisted of a roof, ground, and four walls, which was smaller than what Hassabis made, allowing the subjects to navigate efficiently during the task phase. Four target positions in the corners labeled A, B, C, and D were reachable according to the layouts of decorations on the wall (i.e., clock, panting, door, chair). A birch-colored table was placed in each corner of the room and was immaterial as a cue during navigation. Participants could walk at a realistic speed of 1.9 m/s in the compartments, which is vital in the spatial updating process, as Hassabis et al. (2009) described (Figures 1A,B).

Before scanning, participants immersed themselves in virtual compartments to maximize familiarity with the keyboard controls and were introduced to the navigation task without prior knowledge of matching labels to target positions. They had to reach the target points by the given labels at their own pace. When the participants passed through the incorrect locations, they received feedback from a cross showing on the screen. When the subjects arrived at the designated location, they got a check mark and returned to the virtual room center before the next navigation. Prior to the scanning process, the participants had to meet the criterion performance in a behavioral test in which they had to correctly complete 10 navigation tests in a row in each room to ensure that both layouts had been well mastered. Navigation training took at least 30 min to reach the required performance. Prescanning training was necessary to minimize any learning or novelty effects and stabilize neural activity during retrieval. There was an interval of 1 day between the pretraining task and the formal scanning session. In the retrieval stage, participants entered the room and faced the wall, where a random letter for the navigation target was displayed for 2 s. When they arrived at the targets, the viewpoint transitioned downward so that the identical floor texture occupied the entire field of view. After a 3 s countdown, a new letter was given that was to be followed (Figure 1C). Before proceeding in the scanner, the subject needed to complete as many retrieval tasks as possible in 4 min. The number of task completions and the accuracy of retrieval tasks were gathered for further analysis.

Throughout the scanning, the task proceeded in a block-design manner with a 30 s duration of each block. The task stage alternated with the resting stage in the course of 8 min. No letter prompts and no movements were made in the rest phase, and random perspectives were displayed. The subjects were requested to press buttons at the same frequency as the task and continue to watch the screen until the prompt letter appeared in the center of the screen (Figure 1C). The two stages were matched in terms of visual content and hand movements to the maximum extent.

### Image Preprocessing

The fMRI data were preprocessed using Statistical Parametric Mapping (SPM12)^2^ with the following steps: realignment (correction for head motion-induced intervolume displacement), normalization to the Montreal Neurological Institute (MNI) space using the unified normalization–segmentation procedure *via* the structural images, and spatial smoothing using a Gaussian kernel of 5 mm full-width at half-maximum (FWHM). Default high-pass temporal filtering (1/128 Hz cut-off) in SPM12 was applied to remove low-frequency noise and signal drifts from each voxel’s fMRI time course.

### General Linear Model

The rationale behind the localization of brain regions associated with spatial memory is that the amplitude of such responses depends on the specific spatial task. To formally test this hypothesis, we performed a general linear model (GLM) analysis to identify brain areas where neural activity correlates with spatial memory tasks by contrasting the retrieval state (task phase) and non-retrieval state (rest phase). The two states were modeled as separate regressors in GLM. Six head motion parameters (estimated from the realignment step during fMRI data preprocessing) were included as covariates in the GLM. The contrast maps corresponding to the retrieval state (task phase) minus the non-retrieval state (rest phase) in the first-level analysis were further entered into a second-level one-sample *T*-test to obtain group-level results. The significance level was set at *p* < 0.001 at the voxel level, corrected to *p* < 0.05 using family-wise error (FWE) at the cluster level. Averaged beta values within significantly activated clusters were extracted and correlated with the behavior scores (i.e., retrieval completed times and retrieval accuracy).

### Multivariate Pattern Analysis

MVPA of fMRI signals has recently gained popularity in the neuroimaging community. MVPA is considered a sensitive method to recognize the variation in brain activation and was employed in our analysis. MVPA is a machine learning technique that uses a pattern classifier (Mur et al., 2009; Pereira et al., 2009) to identify the representational content of the neural responses elicited by spatial memory retrieval. In contrast with univariate analyses that detect regional averaged signals and consider a single voxel or a single region of interest (ROI) at a time, MVPA analyzes the spatial pattern of fMRI signals across all voxels within a predefined area. That is, MVPA detects condition-specific patterns of activity across many voxels at once. Whereas GLM directly compares differences in signal amplitude on a voxel-by-voxel basis, MVPA projects samples composed of multiple voxels from each condition of interest into a high-dimensional space and searches for the boundary between the samples from two or more conditions (Mur et al., 2009). MVPA is usually more sensitive than conventional univariate analysis (i.e., GLM) in revealing differences in brain activity between experimental conditions because it offers a powerful solution to the problem of multiple comparisons. It performs a joint analysis of patterns of activity distributed across multiple voxels. In the current study, we applied both within-subject and between-subject MVPAs to detect spatial memory retrieval neural responses. All analyses were conducted using custom scripts written in MATLAB (MathWorks, Natick, MA, United States) in combination with LibSVM implementation of the linear support vector machine (SVM)^3^ using a linear kernel. The parameters of the SVM were set to their default values. In both the within-subject and the between-subject MVPA, we only distinguished between navigational and non-navigational states. If there was no specific information, the average classification accuracy was 50%.

#### Within-Subject MVPA

For within-subject MVPA, the detailed procedures were as follows: for each subject, (1) the averaged BOLD signal of each trial was calculated and labeled as the task stage and the rest stage, (2) the MVPAs were performed in each brain defined by the Brainnetome Atlas^4^ (Fan et al., 2016) and cerebellum templates in Automated Anatomical Labeling (AAL) atlas (Tzourio-Mazoyer et al., 2002), and for each brain region, the BOLD signals of all rest and task trials were extracted and used as features for classification. Leave-one-out cross-validation (LOOCV) was conducted, and the average classification accuracy for each subject was obtained. (3) Subsequently, the accuracy of all subjects within each brain region was fed into one-sample *T*-tests separately to generate a T value brain map. The corresponding *p*-values were corrected by Bonferroni correction (*p* < 0.05/272).

#### Between-Subject MVPA

For between-subject MVPA, the detailed procedures were as follows: (1) the beta maps generated by GLM analyses were labeled as the task stage and the rest stage, (2) MVPAs were performed in each brain defined by the Brainnetome Atlas, and for each brain region, the beta values of all rest and task maps across subjects were extracted and used as features for classification. LOOCV was conducted to obtain group-level classification accuracy. The statistical significance of the classification model was determined by a permutation test (*n* = 1,000) and corrected for multiple comparisons (*p* < 0.05, corrected for FWE) using an in-house MATLAB (R2017a) script. Briefly, in each permutation step, after randomly shuffling the labels of all beta maps, SVM models were performed to generate corresponding classification accuracies in brain regions defined by the Brainnetome Atlas and cerebellum templates in the AAL Atlas, and the maximal classification accuracy across all regions was selected. This procedure was repeated 1,000 times and resulted in 1,000 maximal classification accuracies used to generate the null distribution for calculating the *p*-value of each brain region. Note that because the null distribution was generated using the maximal classification accuracies across all brain regions, the resultant *p*-values were automatically corrected for FWE (Nichols and Hayasaka, 2003).

## Results

### Behavioral Results

All 20 subjects successfully completed the test. In the behavioral research phase, the average accuracy of retrieval tasks within 4 min was 94.52% (*SD* = 5.77), and the average number of task completions was 26.65 (*SD* = 3.13).

### GLM Results and Behavioral Relevance

**FIGURE 2:**
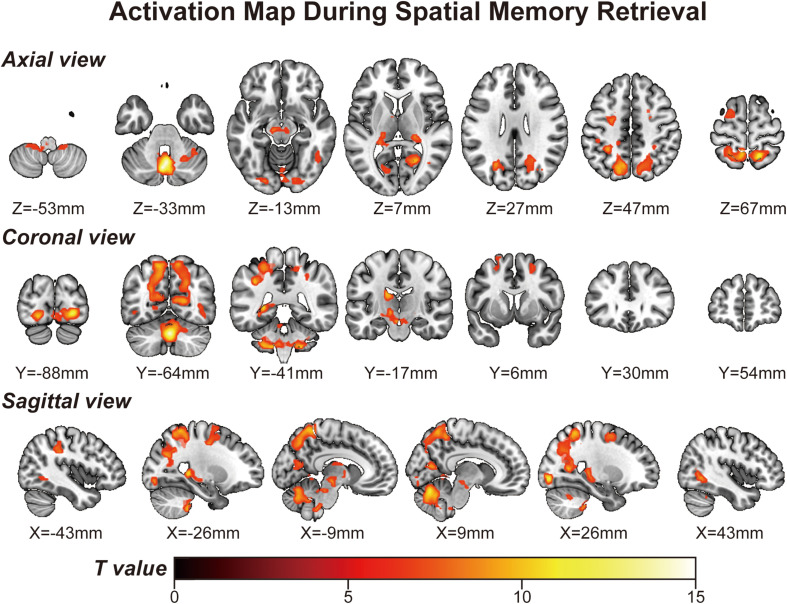
Activation map obtained from the “navigational vs. non-navigational” state (at *p* < 0.001, corrected by FWE for *p* < 0.05 at the cluster level). The hot bar at the bottom labels T value from 0 to 15. Numbers represent X (sagittal view), Y (coronal view), and Z (axial view) coordinates in the MNI space.

**FIGURE 3:**
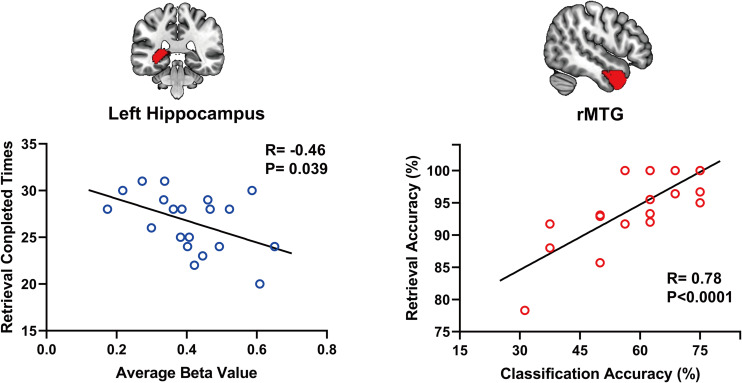
The beta value in the left hippocampus shows a significant negative correlation (*r* = 0.46, *p* = 0.039) with the number of task completions **(left)**. Within-subject classification accuracies positively correlated with the spatial memory retrieval accuracy (*r* = 0.775, *p* = 0.0079) in the rMTG **(right)**.

Using a voxelwise GLM analysis model to determine the effect of spatial memory retrieval, we found that the amplitude of fMRI responses was associated with spatial memory retrieval (at *p* < 0.001, FWE correction corrected for *p* < 0.05 at the cluster level) in widespread brain areas, including the bilateral hippocampi, bilateral frontal superior gyri, bilateral superior parietal lobule (SPL), bilateral occipital lobe, and cerebellum. The detailed information can be found in Table 1. The spatial distribution of these brain regions can be found in Figure 2. In the current study, significantly activated brain regions were the core parts often elicited by spatial memory retrieval (Nilsson et al., 2013; Reynolds et al., 2019). Subsequently, the correspondences between the amplitude brain responses and the behavior performance were detected by performing Pearson correlation analysis. The average beta values of the abovementioned clusters were extracted and correlated with the behavior performance (i.e., completion times and accuracy). The beta value in the left hippocampus showed a significant negative correlation (*r* = 0.46, *p* = 0.039) with the number of task completions quantified by behavior assessment (Figure 3, left).

**TABLE 1:** Detailed information about activation areas in GLM analysis.

Cluster region	Cluster size (voxels)	Peak intensity (*T*-value)	Peak MNI coordinates		
			**X**	**Y**	**Z**

Cluster 1: bilateral parieto-occipital lobe					
Right superior parietal lobule/right precuneus	349	13.63	15	−57	63
Left superior parietal lobule/left precuneus	574	11.63	−17	−57	58
Right calcarine	168	11.03	25	−60	8
Left calcarine	149	7.96	0	−76	8
Cluster 2: cerebellum					
Right cerebellum	482	9.75	26	−35	−44
Left cerebellum	350	10.39	−24	−35	−45
Vermis	141	15.36	−3	−69	−33
Cluster 3: left frontotemporal lobe					
Left superior/middle frontal gyrus/left precentral gyrus	143	9.2866	−24	−9	45
Left hippocampus/left thalamus	112	13.7691	−30	−36	3
Cluster 4: left inferior occipital gyrus	73	10.6014	−21	−87	−9
Cluster 5: right inferior temporal gyrus	69	9.6844	48	−60	−9
Cluster 6: right hippocampus	94	12.1545	27	−27	0
Cluster 7: right superior frontal gyrus	79	8.513	24	3	57

### Within-Subject MVPA

**FIGURE 4:**
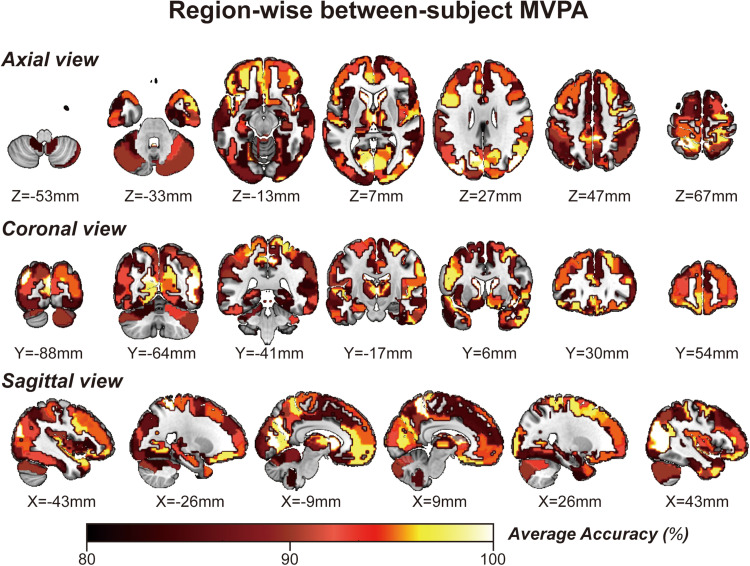
Whole-brain regionwise within-subject MVPA result obtained from “navigational vs. non-navigational” state classification. The hot bar at the bottom indicates percentage accuracy values. Numbers represent X (sagittal view), Y (coronal view), and Z (axial view) coordinates in the MNI space.

Considering GLM restriction, the lack of scrutinization of distribution patterns and information mining toward small sample data, we performed MVPA to detect the neural responses elicited by spatial memory retrieval. We conducted within-subject MVPA in brain regions defined by the Brainnetome Atlas using the average BOLD signal of each trial. We found that widespread brain regions showed high classification accuracies between the task and rest stages (corrected by Bonferroni correction) (Figure 4). The average classification accuracy of each brain region is presented in Table 2. Brain regions with accuracies higher than 80% included two subregions in the right lateral occipital cortex (LOcC), two subregions in the left SPL, one subregion in the right SPL, and two subregions in the medioventral occipital cortex (MVOcC) according to the Brainnetome Atlas. Detailed information on all corrected subregions is shown in Supplementary Table 1. Moreover, we found that the within-subject classification accuracies positively correlated with the spatial memory retrieval accuracy (*r* = 0.775, *p* = 0.0079; Figure 3, right) in the rostral area of the right middle temporal gyrus (rMTG).

**TABLE 2:** Mean accuracy from between-subject and within-subject MVPAs in each corrected region.

Gyrus	Between-subject Mean accuracy (%)			Within-subject Mean accuracy (%)		
	Bilateral	Left	Right	Bilateral	Left	Right
Superior parietal lobule	94.29	95.00	93.75	78.00	80.00	76.00
Medioventral occipital cortex	95.00	95.63	94.38	76.72	74.50	78.94
Lateral occipital cortex	91.59	89.17	94.50	78.46	76.51	80.42
Precuneus	94.06	95.00	93.13	73.75	73.91	73.59
Paracentral lobule	97.50	95.00	100.00	68.13	68.13	68.13
Postcentral gyrus	94.29	95.00	93.75	71.25	75.52	66.98
Superior frontal gyrus	95.36	93.93	96.79	69.31	68.33	70.78
Precentral gyrus	93.13	93.13	93.13	70.94	71.93	68.96
Vermis	91.25			71.98		
Inferior parietal lobule	91.88	90.83	92.92	70.74	70.10	71.50
Inferior temporal gyrus	90.75	91.88	90.00	70.44	71.04	69.53
Middle frontal gyrus	92.68	92.14	93.21	67.86	69.11	66.61
Basal ganglia	95.42	95.00	95.83	64.94	64.22	65.42
Orbital gyrus	93.75	95.83	91.67	64.79	65.21	64.38
Middle temporal gyrus	88.44	85.00	91.88	69.84	68.13	71.56
Fusiform gyrus	85.00	87.50	82.50	72.66	75.16	70.16
Cingulate gyrus	91.56	90.63	92.50	65.86	66.80	64.92
Cerebellum	86.50	85.00	88.00	70.66	71.70	69.86
Thalamus	92.08	92.50	91.67	64.95	64.06	65.83
Superior temporal gyrus	93.33	92.50	94.38	62.97	62.19	63.75
Inferior frontal gyrus	92.50	92.00	93.00	62.19	62.27	62.03
Insular gyrus	92.27	89.58	95.50	61.56	62.19	61.25
Posterior superior temporal sulcus	83.75	–	83.75	64.69	65.47	63.13
Hippocampus	92.50	93.75	90.00	None		
Parahippocampal gyrus	85.50	82.50	86.25	None		
Amygdala	83.75	–	83.75	None		

*None, no subregion in this area was corrected. The vermis cannot be divided into the left and right parts.*

### Between-Subject MVPA

**FIGURE 5:**
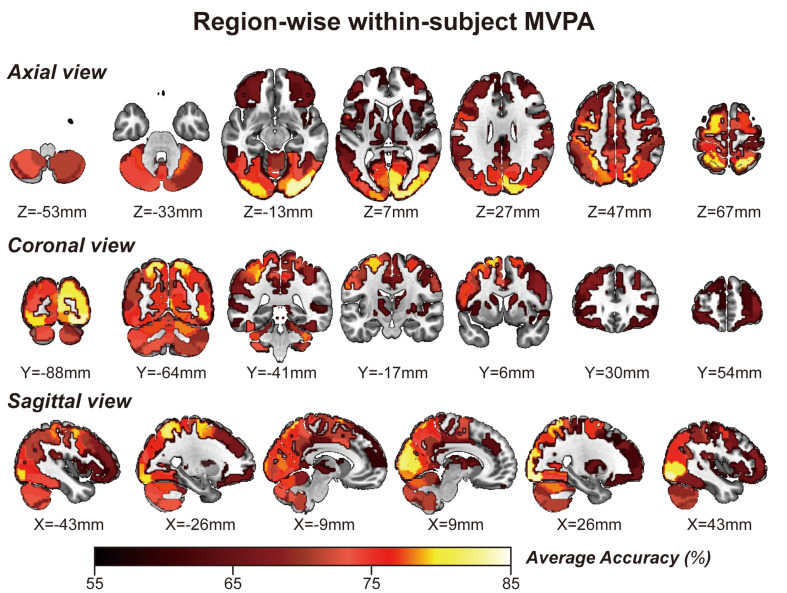
Whole-brain regionwise between-subject MVPA result obtained from “navigational vs. non-navigational” state classification. The hot bar at the bottom indicates percentage accuracy values. Numbers represent X (sagittal view), Y (coronal view), and Z (axial view) coordinates in the MNI space.

By applying regionwise between-subject MVPA, we found that numerous regions showed high classification accuracy between task- and rest-stage beta maps. These brain regions are mainly located in the paracentral lobule, basal ganglia, superior frontal gyrus, MVOcC, and SPL (Figure 5). The arithmetic average classification accuracy of each brain region is presented in Table 2. Detailed information can be found in Supplementary Table 2. These brain regions mainly overlapped with our previous results.

## Discussion

The present results indicate that the brain’s vast regions are associated with spatial memory retrieval in univariate and multivariate ways. Spatial memory recognition therefore depends on the whole brain, not just the MTL structures. In the behavioral analysis, the amplitude of average fMRI signals in the left hippocampus showed a significant negative correlation with the number of task completions in a limited time. In addition, task retrieval accuracy positively correlates with the classification accuracy given by the patterns of the rMTG rostral areas in the Brainnetome Atlas. Therefore, MVPA-based spatial memory analysis should be extended from the hippocampus or MTL structures to the whole-brain level to deepen our understanding of spatial memory.

### Multivariate-Based Brain Map in Spatial Memory

The increasing application of MVPA brings sensitivity to fluctuations in multivariate information and offers the possibility to attach further details in spatial memory. Chadwick et al. (2012) previously reviewed their four published MVPA studies that specifically focused on the MTL, including spatial information recognition (Hassabis et al., 2009), individual episodic memory discrimination (Chadwick et al., 2010), identifying the overlapping information in episodes (Chadwick et al., 2011), and decoding overlapping scene representations (Bonnici et al., 2012). Their experiments convincingly highlight the potential utility of MVPA in hippocampal analyses. Additionally, recent publications have significantly improved hippocampus-specific analysis of spatial memory (Hassabis et al., 2009; Nielson et al., 2015; Deuker et al., 2016). Hassabis et al. (2009) demonstrated that the hippocampal neuron population represented precise spatial positions, whereas voxels in the parahippocampal gyrus expressed environmental information in two virtual cubical spaces. We also used this paradigm in our research to distinguish the differences between navigational and non-navigational states instead of spatial location or environmental information. Deuker et al. (2016) introduced a novel experimental paradigm that allowed them to investigate spatial and temporal aspects of memory in a large-scale virtual city. Their results provided a new explanation for a common coding mechanism of episodic and spatial memory, in which the spatiotemporal network was reflected in the hippocampus’s neural patterns. In real life, another report showed that both spatial and temporal contexts were encoded within the hippocampus across various scales of magnitude, up to 1 month in time and 30 km in distance (Nielson et al., 2015). MVPAs enable scientists to decode complex, overlapping, and practical information from voxel patterns in the human hippocampus, whether in rigorous laboratory conditions or in the real world.

Remarkably, the MVPA method primarily focuses on detecting diverse patterns of neural signal changes relating to specific stimuli information in the brain in addition to the BOLD intensity from standard fMRI analyses. Region-specific analyses by MVPA within the hippocampus consolidate its place in spatial memory retrieval and navigation. However, there is less focus on the rest of the brain areas. Thus, it is worth exploring whether there is any difference between the multivariate-based brain map and univariate-based activation map in the spatial memory task. This study aims to identify cortices involving spatial memory across the brain by MVPA, referring to Hassabis’s paradigm (Hassabis et al., 2009). We first present a multivariate-based spatial navigation map consisting mainly of the bilateral hippocampi, bilateral superior frontal gyrus, bilateral SPLs, bilateral occipital lobes, and cerebellum. The univariate (Figure 2) and multivariate (Figures 4, 5) images display a fair visual uniformity in spatial distribution. To show consistency with similar experiments, Neurosynth (Yarkoni et al., 2011), an automated meta-analysis platform^5^, was used to present a navigation-based synthetic activation map (Supplementary Figure 1) from 77 published articles. As in the above three maps (Uncorrected Annotation Univariate map, Figure 2; Multivariate map, Figures 4, 5; Synthetic map, Supplementary Figure 1), brain regions in the “navigation network” beyond the hippocampus mainly overlap with three regions, SPL, MVOcC, and LOcC.

To better illustrate the navigation map, we need to describe the spatial information processing method in detail. Spatial processing is well organized by two hierarchical pathways: the ventral stream (or the “what” pathway) for object vision and the dorsal stream (or the “how” pathway) for spatial vision (Ungerleider and Haxby, 1994; Konen and Kastner, 2008). Kravitz et al. (2011) updated a novel neural framework for visuospatial processing and redefined the dorsal stream’s anatomical origin. Posterior regions of the parietal cortex, including medial portions of the SPL, are critical in the dorsal stream. Much research has provided evidence that some parietal lobe parts encode spatial performance in the human brain (Fein et al., 2009; Gottlieb and Snyder, 2010; Vandenberghe et al., 2012). The SPL plays a central role in spatial functions, such as spatial attention, saccadic eye movements (Gottlieb and Snyder, 2010), and memory tasks (Simons et al., 2010; Vandenberghe et al., 2012). Posterior parietal lesions can also lead to egocentric disorientation (Aguirre and D’Esposito, 1999) and an inability to localize objects in space (Kase et al., 1977). This deficit indicates that the posterior parietal cortex is the source of the egocentric information needed for navigation. In our research, bilateral occipitoparietal cortices were revealed among these three maps (Figures 2, 4, 5) with a high T value in GLM analysis and high accuracy of MVPA classification. These results strengthen the previous conclusion that the posterior parietal cortex is associated with spatial memory in BOLD signal intensity and distribution patterns. However, it is difficult to determine how the three pathways form in the posterior parietal cortex. The distribution pattern within this region may involve the separation of functions, which MVPA might resolve.

The GLM approach, the “gold” standard in fMRI research, is usually referred to as a mass univariate model-based analysis, revealing linear correlations between time course and task design. MVPA is a classification algorithm that captures subtle differences in informative voxel distributions between various conditions based on SVM in general. Both methods were adopted in this research to compare the distribution of multivariate and univariate information. In our maps, although the multivariate and univariate information overlapped for the most part, there were slight differences as well. Classification accuracy within the hippocampus is lower than that calculated from the SPL, MVOcC, and LOcC, especially in within-subject MVPA, in which the classification accuracy fails to pass the correction. The decision whether to navigate is likely embodied in these areas. Further detailed experimental designs are needed to compare the representation of navigation decisions in these brain regions.

### Behavioral Analyses in the Hippocampus and rMTG

The MTL structures, particularly the hippocampus, showed enhanced brain activity during navigation (Epstein et al., 2017). We found the same neural signaling alterations in the hippocampus (Figure 2 and Table 1). In the behavior analysis, we noticed that the beta value of the left hippocampus showed a significant negative correlation (*r* = 0.46, *p* = 0.039) with the number of task completions in a limited time. Because all subjects underwent multiple exercises before the scanning, we assume that lower neural activity was required for well-trained subjects than for others to achieve the same performance. The reduction in neural activity caused by repeated stimuli is the robust cortical activity associated with experience (Vannini et al., 2013). In studies of episodic memory, repetition suppression has been observed in the hippocampus and adjacent brain regions in the MTL, suggesting that suppression may reflect successful encoding or consolidation (Gonsalves et al., 2005; Rand-Giovannetti et al., 2006; Suzuki et al., 2011). A significant number of studies using two-dimensional images as experimental materials have produced the same conclusion, although a similar effect in a three-dimensional environment has not yet been confirmed.

We first report that classification accuracy based on the patterns of the rMTG rostral areas in the Brainnetome Atlas showed a remarkably significant correlation with task performance by using MVPA. Under the strictest correction, the rostral areas of the rMTG, located in the anterior temporal lobe, showed a high Pearson correlation coefficient (*r* = 0.7750), which suggested that potential neural function correlated with spatial memory retrieval. A previous study indicated that anterior temporal lobe resection might impair memory function, typically visual memory following right anterior temporal lobe resection (Bonelli et al., 2013). Patients who undergo from right anterior temporal resections demonstrate deficits of memory for locations in both two-dimensional and three-dimensional performances (Diaz-Asper et al., 2006). We suggest that the rostral areas of the rMTG may serve as an essential component of the parieto-medial temporal pathway and have a vital function in visuospatial retrieval.

## Limitations

Gender differences were frequently discussed in previous studies (Persson et al., 2013; Spets et al., 2019) that showed that men have better performance in spatial memory tasks and different patterns of cortical activity. To better elucidate how spatial memory is encoded in the brain, we recruited only male subjects to ensure homogeneity. However, sex comparisons should be a significant component of further studies. With regard to data continuity and comparability, we collected only behavioral data from the MRI unit. Further analysis should be performed using behavioral data obtained both inside and outside the MRI unit.

## Conclusion

In summary, whole-brain maps of spatial memory retrieval in cubical space were generated using GLM and MVPA in our study. Certain distribution patterns in these regions, the posterior parietal cortex and rMTG, are specific to spatial memory retrieval. These results show global and regional effects in the brain during spatial memory retrieval. MVPA provides assistance in obtaining more information about spatial memory in the brain.

## Data Availability Statement

The raw data supporting the conclusions of this article will be made available by the authors, without undue reservation.

## Ethics Statement

The studies involving human participants were reviewed and approved by the Medical Ethics Committee of Tianjin Medical University General Hospital. The patients/participants provided their written informed consent to participate in this study. Written informed consent was obtained from the individual(s) for the publication of any potentially identifiable images or data included in this article.

## Author Contributions

JG wrote the main manuscript text. JG and RZ performed the data analyses. KZ and JZ designed the experiments. JZ, YLin, and SY conducted the experiments. JZ and YLia was responsible for data collection. KZ, WQ, and XY supervised the experimental framework. KZ and XY supervised the analyses. All authors have read and agreed to the published version of the manuscript.

## Conflict of Interest

The authors declare that the research was conducted in the absence of any commercial or financial relationships that could be construed as a potential conflict of interest.

## Footnotes

1 https://unity3d.com/

2 https://www.fil.ion.ucl.ac.uk/spm/

3 www.csie.ntu.edu.tw/∼cjlin/libsvm

4 http://atlas.brainnetome.org

5 www.neurosynth.org
