# Supplementary material for: Decoding Spatial Memory Retrieval in Cubical Space Using fMRI Signals
Source: Front Neural Circuits. 2021 May 18;15:624352. doi: 10.3389/fncir.2021.624352 (PMC8168467; doi:10.3389/fncir.2021.624352)

Supplementary Tabel 1.

Whole_brain Within_subject MVPA accuracy in each corrected region

Gyrus	Mean Accuracy (%)	Label in template（Left）	Accuracy (%)	Label in template（Right）	Accuracy (%)
LOcC, Lateral Occipital Cortex,	78.46	LOcC_L_4_4	79.38	LOcC_R_4_4	83.44
		LOcC _L_4_3	78.44	LOcC _R_4_3	82.50
		LOcC _L_2_2	77.50	LOcC_R_4_1	80.00
		LOcC _L_4_2	75.63	LOcC _R_4_2	80.00
		LOcC _L_2_1	74.69	LOcC _R_2_1	80.00
		LOcC_L_4_1	73.44	LOcC _R_2_2	76.56
SPL, Superior Parietal Lobule	78.00	SPL_L_5_5	81.88	SPL_R_5_1	81.56
		SPL_L_5_1	81.56	SPL_R_5_2	79.06
		SPL_L_5_4	79.38	SPL_R_5_5	77.81
		SPL_L_5_3	79.06	SPL_R_5_4	72.81
		SPL_L_5_2	78.13	SPL_R_5_3	68.75
MVOcC, MedioVentral Occipital Cortex	76.72	MVOcC _L_5_2	77.81	MVOcC _R_5_3	81.88
		MVOcC _L_5_5	76.56	MVOcC _R_5_2	80.63
		MVOcC _L_5_1	74.69	MVOcC _R_5_1	78.13
		MVOcC _L_5_4	72.50	MVOcC _R_5_4	77.50
		MVOcC _L_5_3	70.94	MVOcC _R_5_5	76.56
Pcun, Precuneus	73.75	PCun_L_4_3	76.56	PCun_R_4_2	75.94
		PCun_L_4_1	75.31	PCun_R_4_1	74.69
		PCun_L_4_2	73.44	PCun_R_4_3	73.75
		PCun_L_4_4	70.31	PCun_R_4_4	70.00
FuG, Fusiform Gyrus	72.66	FuG_L_3_2	76.56	FuG_R_3_2	72.81
		FuG_L_3_3	73.75	FuG_R_3_3	67.50
Vermis	71.98	Vermis_7		75.94	
		Vermis_6		75.31	
		Vermis_8		74.38	
		Vermis_9		70.63	
		Vermis_4_5		70.00	
		Vermis_3		65.63	
PoG, Postcentral Gyrus	71.25	PoG_L_4_3	78.13	PoG_R_4_3	71.25
		PoG_L_4_4	75.63	PoG_R_4_4	65.31

		PoG_L_4_1	72.81	PoG_R_4_1	64.38
PrG, Precentral Gyrus	70.94	PrG_L_6_2	79.06	PrG_R_6_2	76.56
		PrG_L_6_6	76.88	PrG_R_6_3	65.31
		PrG_L_6_3	71.25	PrG_R_6_6	65.00
		PrG_L_6_4	70.94		
		PrG_L_6_1	70.31		
		PrG_L_6_5	63.13		
IPL, Inferior Parietal Lobule	70.74	IPL_L_6_1	75.31	IPL_R_6_1	75.31
		IPL_L_6_2	71.25	IPL_R_6_2	75.00
		IPL_L_6_5	70.94	IPL_R_6_5	70.00
		IPL_L_6_3	70.63	IPL_R_6_3	69.06
		IPL_L_6_6	67.50	IPL_R_6_6	68.13
		IPL_L_6_4	65.00		
Cerebellum	70.66	Cerebellum_6_L	76.88	Cerebellum_6_R	77.50
		Cerebellum_Crus1_L	74.06	Cerebellum_Crus2_R	71.56
		Cerebellum_Crus2_L	72.81	Cerebellum_8_R	70.94
		Cerebellum_8_L	72.50	Cerebellum_9_R	70.63
		Cerebellum_9_L	70.63	Cerebellum_4_5_R	69.69
		Cerebellum_4_5_L	67.50	Cerebellum_Crus1_R	69.38
		Cerebellum_7b_L	67.50	Cerebellum_7b_R	69.38
				Cerebellum_10_R	67.50
				Cerebellum_3_R	62.19
ITG, Inferior Temporal Gyrus	70.44	ITG_L_7_2	76.25	ITG_R_7_5	73.44
		ITG_L_7_5	75.31	ITG_R_7_2	65.63
		ITG_L_7_6	61.56		
MTG, Middle Temporal Gyrus	69.84	MTG_L_4_3	68.13	MTG_R_4_3	71.56
SFG, Superior Frontal Gyrus	69.31	SFG_L_7_4	79.38	SFG_R_7_4	76.88
		SFG_L_7_5	71.25	SFG_R_7_5	72.19
		SFG_L_7_1	70.94	SFG_R_7_1	70.00
		SFG_L_7_2	66.56	SFG_R_7_6	64.06
		SFG_L_7_3	62.19		
		SFG_L_7_7	59.69		
PCL, Paracentral Lobule	68.13	PCL_L_2_1	68.13	PCL_R_2_1	68.44

		PCL_L_2_2	68.13	PCL_R_2_2	67.81
MFG, Middle Frontal Gyrus	67.86	MFG_L_7_6	77.81	MFG_R_7_6	75.00
		MFG_L_7_2	70.31	MFG_R_7_1	66.56
		MFG_L_7_5	68.13	MFG_R_7_2	65.94
		MFG_L_7_4	66.88	MFG_R_7_7	65.63
		MFG_L_7_1	66.25	MFG_R_7_4	65.00
		MFG_L_7_7	65.31	MFG_R_7_3	61.56
CG, Cingulate Gyrus	65.86	CG_L_7_1	64.38	CG_R_7_4	66.88
		CG_L_7_5	73.44	CG_R_7_6	66.25
		CG_L_7_4	65.31	CG_R_7_5	65.31
		CG_L_7_6	64.06	CG_R_7_1	61.25
Tha, Thalamus	64.95	Tha_L_8_6	65.00	Tha_R_8_1	66.56
		Tha_L_8_8	64.38	Tha_R_8_7	65.63
		Tha_L_8_5	62.81	Tha_R_8_5	65.31
BG, Basal Ganglia	64.94	BG_L_6_2	65.00	BG_R_6_5	66.25
		BG_L_6_5	63.44	BG_R_6_2	65.63
				BG_R_6_6	64.38
OrG, Orbital Gyrus	64.79	OrG_L_6_3	66.25	OrG_R_6_2	65.31
		OrG_L_6_2	64.69	OrG_R_6_6	64.69
		OrG_L_6_6	64.69	OrG_R_6_3	63.13
pSTS, posterior Superior Temporal Sulcus	64.69	pSTS_L_2_2	65.94	pSTS_R_2_1	63.13
		pSTS_L_2_1	65.00		
STG, Superior Temporal Gyrus	62.97	STG_L_6_4	62.19	STG_R_6_3	63.75
IFG, Inferior Frontal Gyrus	62.19	IFG_L_6_5	64.69	IFG_R_6_5	64.38
		IFG_L_6_1	63.44	IFG_R_6_4	59.69
		IFG_L_6_2	62.19		
		IFG_L_6_4	58.75		
INS, Insular Gyrus	61.56	INS_L_6_6	62.19	INS_R_6_3	62.81
				INS_R_6_4	59.69

Note. No subregion in amygdala, hippocampus and parahippocampus gyrus is corrected in bilateral hemishphere. The vermis cannot be divided into left and right parts.

Supplementary Tabel 2.

Whole_brain Between_subject MVPA accuracy in each corrected region

Gyrus	Mean Accuracy (%)	Label in template（Left）	Accuracy (%)	Label in template（Right）	Accuracy (%)
PCL, Paracentral Lobule	97.50	PCL_L_2_1	95.0	PCL_R_2_1	100.0
		PCL_L_2_2	95.0	PCL_R_2_2	100.0
BG, Basal Ganglia	95.42	BG_L_6_1	100.0	BG_R_6_5	100.0
		BG_L_6_5	100.0	BG_R_6_3	95.0
SFG, Superior Frontal Gyrus	95.36	SFG_L_7_1	100.0	SFG_R_7_1	100.0
		SFG_L_7_6	100.0	SFG_R_7_5	100.0
		SFG_L_7_2	95.0	SFG_R_7_6	100.0
		SFG_L_7_3	95.0	SFG_R_7_7	100.0
		SFG_L_7_7	95.0	SFG_R_7_3	95.0
		SFG_L_7_4	87.5	SFG_R_7_4	92.5
		SFG_L_7_5	85.0	SFG_R_7_2	90.0
MVOcC, MedioVentral Occipital Cortex	95.00	MVOcC _L_5_2	100.0	MVOcC _R_5_2	97.5
		MVOcC _L_5_4	97.5	MVOcC _R_5_5	95.0
		MVOcC _L_5_5	97.5	MVOcC _R_5_3	92.5
		MVOcC _L_5_3	87.5	MVOcC _R_5_4	92.5
SPL, Superior Parietal Lobule	94.29	SPL_L_5_4	100.0	SPL_R_5_4	97.5
		SPL_L_5_1	95.0	SPL_R_5_3	95.0
		SPL_L_5_3	90.0	SPL_R_5_2	92.5
				SPL_R_5_1	90.0
PoG, Postcentral Gyrus	94.29	PoG_L_4_3	95.0	PoG_R_4_3	97.5
		PoG_L_4_4	95.0	PoG_R_4_4	97.5
		PoG_L_4_1	90.0	PoG_R_4_2	95.0
		PoG_L_4_2	90.0		
Pcun, Precuneus	94.06	PCun_L_4_3	100.0	PCun_R_4_2	100.0
		PCun_L_4_2	97.5	PCun_R_4_3	97.5
		PCun_L_4_4	92.5	PCun_R_4_4	90.0
		PCun_L_4_1	90.0	PCun_R_4_1	85.0
OrG, Orbital Gyrus	93.75	OrG_L_6_2	100.0	OrG_R_6_1	95.0
		OrG_L_6_5	100.0	OrG_R_6_3	95.0
		OrG_L_6_3	97.5	OrG_R_6_4	95.0

		OrG_L_6_4	97.5	OrG_R_6_2	90.0
		OrG_L_6_1	90.0	OrG_R_6_5	90.0
		OrG_L_6_6	90.0	OrG_R_6_6	85.0
STG, Superior Temporal Gyrus	93.33	STG_L_6_5	97.5	STG_R_6_5	100.0
		STG_L_6_1	95.0	STG_R_6_1	95.0
		STG_L_6_4	92.5	STG_R_6_4	92.5
		STG_L_6_3	90.0	STG_R_6_3	90.0
		STG_L_6_6	87.5		
PrG, Precentral Gyrus	93.13	PrG_L_6_3	95.0	PrG_R_6_2	97.5
		PrG_L_6_4	95.0	PrG_R_6_3	95.0
		PrG_L_6_2	92.5	PrG_R_6_5	95.0
		PrG_L_6_6	90.0	PrG_R_6_4	85.0
MFG, Middle Frontal Gyrus	92.68	MFG_L_7_2	95.0	MFG_R_7_5	100.0
		MFG_L_7_4	95.0	MFG_R_7_1	95.0
		MFG_L_7_5	95.0	MFG_R_7_3	95.0
		MFG_L_7_7	95.0	MFG_R_7_4	92.5
		MFG_L_7_3	92.5	MFG_R_7_7	92.5
		MFG_L_7_1	87.5	MFG_R_7_6	90.0
		MFG_L_7_6	85.0	MFG_R_7_2	87.5
IFG, Inferior Frontal Gyrus	92.50	IFG_L_6_1	90.0	IFG_R_6_1	95.0
		IFG_L_6_2	95.0	IFG_R_6_2	97.5
		IFG_L_6_3	97.5	IFG_R_6_3	100.0
		IFG_L_6_5	82.5	IFG_R_6_4	87.5
		IFG_L_6_6	95.0	IFG_R_6_6	85.0
Hipp, Hippocampus	92.50	Hipp_L_2_1	95.0	Hipp_R_2_2	90.0
		Hipp_L_2_2	92.5		
INS, Insular Gyrus	92.27	INS_L_6_2	100.0	INS_R_6_2	100.0
		INS_L_6_4	92.5	INS_R_6_4	100.0
		INS_L_6_5	92.5	INS_R_6_6	95.0
		INS_L_6_3	85.0	INS_R_6_5	92.5
		INS_L_6_6	85.0	INS_R_6_1	90.0
		INS_L_6_1	82.5		
Tha, Thalamus	92.08	Tha_L_8_1	97.5	Tha_R_8_4	100.0

		Tha_L_8_4	97.5	Tha_R_8_7	100.0
		Tha_L_8_7	97.5	Tha_R_8_8	97.5
		Tha_L_8_5	90.0	Tha_R_8_1	85.0
		Tha_L_8_8	87.5	Tha_R_8_2	85.0
		Tha_L_8_6	85.0	Tha_R_8_5	82.5
IPL, Inferior Parietal Lobule	91.88	IPL_L_6_1	100.0	IPL_R_6_1	100.0
		IPL_L_6_5	92.5	IPL_R_6_5	97.5
		IPL_L_6_3	90.0	IPL_R_6_6	95.0
		IPL_L_6_6	90.0	IPL_R_6_2	90.0
		IPL_L_6_4	87.5	IPL_R_6_4	90.0
		IPL_L_6_2	85.0	IPL_R_6_3	85.0
LOcC, lateral Occipital Cortex	91.59	LOcC_L_4_2	95.0	LOcC_R_4_2	100.0
		LOcC_L_4_1	92.5	LOcC_R_4_3	97.5
		LOcC_L_2_1	90.0	LOcC_R_4_1	95.0
		LOcC_L_4_4	87.5	LOcC_R_2_1	95.0
		LOcC_L_4_3	85.0	LOcC_R_4_4	85.0
		LOcC_L_2_2	85.0		
CG, Cingulate Gyrus	91.56	CG_L_7_3	97.5	CG_R_7_2	95.0
		CG_L_7_7	97.5	CG_R_7_6	95.0
		CG_L_7_6	85.0	CG_R_7_7	95.0
		CG_L_7_2	82.5	CG_R_7_3	85.0
Vermis	91.25	Vermis_10		100.0	
		Vermis_3		90.0	
		Vermis_7		87.5	
		Vermis_9		87.5	
ITG, Inferior Temporal Gyrus	90.75	ITG_L_7_4	95.0	ITG_R_7_1	97.5
		ITG_L_7_5	92.5	ITG_R_7_7	92.5
		ITG_L_7_7	92.5	ITG_R_7_3	90.0
		ITG_L_7_1	87.5	ITG_R_7_4	90.0
				ITG_R_7_5	85.0
				ITG_R_7_6	85.0
MTG, Middle Temporal Gyrus	88.44	MTG_L_4_1	87.5	MTG_R_4_3	100.0
		MTG_L_4_3	85.0	MTG_R_4_1	92.5

		MTG_L_4_4	85.0	MTG_R_4_2	90.0
		MTG_L_4_2	82.5	MTG_R_4_4	85.0
Cerebellum	86.50	Cerebellum_Crus1_L	90.0	Cerebellum_6_R	92.5
		Cerebellum_4_5_L	87.5	Cerebellum_Crus1_R	90.0
		Cerebellum_3_L	82.5	Cerebellum_Crus2_R	90.0
		Cerebellum_6_L	82.5	Cerebellum_9_R	85.0
		Cerebellum_9_L	82.5	Cerebellum_7b_R	82.5
PhG, Parahippocampal Gyrus	85.50	PhG_L_6_3	82.5	PhG_R_6_4	90.0
				PhG_R_6_5	87.5
				PhG_R_6_1	85.0
				PhG_R_6_6	82.5
FuG, Fusiform Gyrus	85.00	FuG_L_3_3	92.5	FuG_R_3_1	82.5
		FuG_L_3_2	82.5	FuG_R_3_2	82.5
pSTS, posterior Superior Temporal Sulcus	83.75	None		pSTS_R_2_1	85.0
				pSTS_R_2_2	82.5
Amyg, Amygdala	83.75	None		Amyg_R_2_1	85.0
				Amyg_R_2_2	82.5

Note. None, no subregion. The vermis cannot be divided into left and right parts.

Supplementary Figure 1. An automated neurosynth meta-analysis based on the term “navigation” obtained, corrected using a false discovery rate (FDR) approach, with an expected FDR of 0.01. The meta-analysis was performed by automatically identifying all studies in the Neurosynth database that loaded highly on the term, and then performing meta-analyses to identify brain regions that were consistently or preferentially reported in the tables of those studies. The hot bar at the bottom labels Z score from 0 to 7.5. Numbers represent X (sagittal view), Y (coronal view), and Z (axial view) coordinates in MNI space.

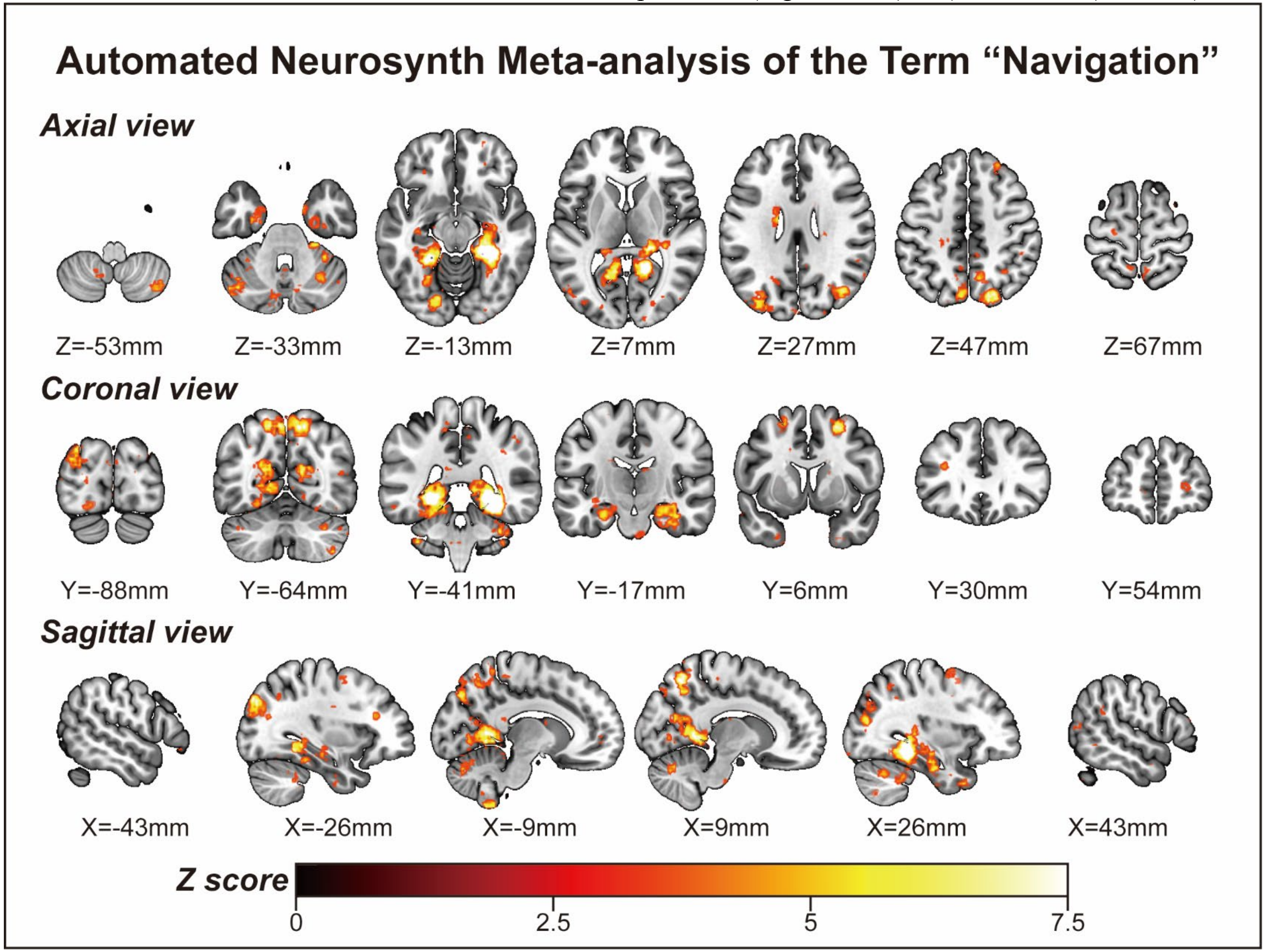

Supplement: Supplementary file 1 [file Data_Sheet_1.pdf]
